# Growth patterns during early childhood in children born small for gestational age and moderate preterm

**DOI:** 10.1038/s41598-019-48055-x

**Published:** 2019-08-09

**Authors:** Linda Lindström, Fredrik Ahlsson, Maria Lundgren, Eva Bergman, Erik Lampa, Anna-Karin Wikström

**Affiliations:** 1 Department of Women’s and Children’s Health, Uppsala University, Uppsala, Sweden; 2 Uppsala Clinical Research Center, UCR, Uppsala University, Uppsala, Sweden

**Keywords:** Endocrinology, Endocrine system and metabolic diseases, Paediatrics

## Abstract

Today we lack knowledge if size at birth and gestational age interact regarding postnatal growth pattern in children born at 32 gestational weeks or later. This population-based cohort study comprised 41,669 children born in gestational weeks 32–40 in Uppsala County, Sweden, between 2000 and 2015. We applied a generalized least squares model including anthropometric measurements at 1.5, 3, 4 and 5 years. We calculated estimated mean height, weight and BMI for children born in week 32 + 0, 35 + 0 or 40 + 0 with birthweight 50^th^ percentile (standardized appropriate for gestational age, *s*AGA) or 3^rd^ percentile (standardized small for gestational age, *s*SGA). Compared with children born *s*AGA at gestational week 40 + 0, those born *s*AGA week 32 + 0 or 35 + 0 had comparable estimated mean height, weight and BMI after 3 years of age. Making the same comparison, those born *s*SGA week 32 + 0 or 35 + 0 were shorter and lighter with lower estimated mean BMI throughout the whole follow-up period. Our findings suggest that being born SGA and moderate preterm is associated with short stature and low BMI during the first five years of life. The association seemed stronger the shorter gestational age at birth.

## Introduction

Being born small for gestational age (SGA) is associated with increased risk of metabolic and cardiovascular complications as well as suboptimal cognitive development later in life^1,2^. 85–90% of children born SGA show linear catch-up growth during early childhood and will to a large extent reach normal adult stature^3^. However, catch-up growth may have both negative and positive effects on future health. The risk of obesity and cardiovascular disease increases, whereas cognitive impairment decreases after rapid catch-up growth^4–7^.

Children born preterm are more often born SGA than those born term^8,9^. Birth at gestational week 32–36, referred to as moderate to late preterm birth, accounts for approximately 85% of all preterm births. Despite this, most research on preterm birth involves children born before gestational week 32^10^.

Slow neonatal weight gain is common in preterm born children of all birthweights, and is more pronounced the lower the gestational age^11,12^. Hence, not only children born SGA, but also children born appropriate for gestational age (AGA) but preterm may experience a period of perinatal growth restriction. Rapid catch-up growth seems to promote cognitive development in children born moderate preterm, but the evidence regarding metabolic outcomes is scarce in this group^13,14^.

Deviating postnatal growth is more common in children born SGA with comorbidity and short gestational age^15,16^. Earlier research on postnatal growth in children born preterm often comprises study populations of diverse gestational age and birthweight. With substantial neonatal growth restriction and need of intensive care in the most preterm born infants, potential adverse effects might vary widely within study populations including extreme as well as late preterm births^17^.

In this population-based study we had the opportunity to study postnatal growth patterns until five years of age in almost 42,000 children born between gestational week 32 and 40. We hypothesized that SGA and gestational age at birth may interact on postnatal growth patterns in children born moderate preterm. Further, that body proportions at five years may differ depending on size and gestational age at birth.

## Results

Of the 41,669 children included in the study, 532 (1.3%) were born at 32–34 gestational weeks, 1692 (4.1%) at 35–36 weeks, 11,271 (27.0%) at 37–38 weeks and 28,174 (67.6%) at 39–40 weeks. Birthweight and birth length for gestational age and sex were the lowest in children born moderate preterm (32–34 weeks), with p-value for overall difference between groups < 0.05. Children born in late term pregnancy (39–40 weeks) had the tallest mothers, 1 cm taller mean height than the other groups, p < 0.05. The rate of maternal smoking was the highest in children born late preterm (35–36 weeks). Birthweight and birth length for gestational age and sex were the lowest in children born moderate preterm (32–34 weeks), p < 0.05. In all gestational ages, a large proportion of the children had missing information on breastfeeding at age two months. The characteristics of the study population are summarized in Table 1.

**Table 1:** Characteristics of study population.

	Gestational Age					P-value
	Total (n = 41,669)	32–34 weeks (n = 532)	35–36 weeks (n = 1692)	37–38 weeks (n = 11,271)	39–40 weeks (n = 28,174)	
**Maternal Characteristics**						
Age, years, mean (SD)	30.4 (5.1)	30.0 (5.4)	30.0 (5.3)	30.7 (5.2)	30.3 (5.0)	<0.05
Height, cm, mean (SD)	166.4 (6.4)	165.6 (5.9)	165.8 (6.5)	165.8 (6.4)	166.7 (6.4)	<0.05
missing, n (%)	1324 (3.2%)	24 (4.5%)	65 (3.8%)	361 (3.2%)	874 (3.1%)	
BMI early pregnancy, kg/m^2^, mean (SD)	24.6 (4.5)	25.1 (4.8)	24.7 (4.7)	24.8 (4.8)	24.5 (4.4)	<0.05
missing, n (%)	4003 (9.6%)	56 (10.5%)	174 (10.3%)	1111 (9.9%)	2662 (9.4%)	
Parity, mean (SD)	1.9 (1.0)	1.7 (1.0)	1.8 (1.1)	2.0 (1.1)	1.9 (1.0)	<0.05
Country of birth^a^, n (%)						
Nordic country, n (%)	34,415 (82.6%)	450 (84.6%)	1418 (83.8%)	9196 (81.6%)	23,351 (82.9%)	
Europe, North America, n (%)	1489 (3.6%)	25 (4.7%)	45 (2.7%)	377 (3.3%)	1042 (3.7%)	
other, n (%)	5741 (13.8%)	57 (10.7%)	228 (13.5%)	1688 (15.0%)	3768 (13.4%)	
missing, n (%)	24 (0.1%)	0	1 (0.1%)	10 (0.1%)	13 < 0.1%)	
Level of education, years						<0.05
≤9, n (%)	3753 (9.0%)	48 (9.0%)	179 (10.6%)	1107 (9.8%)	2419 (8.6%)	
10–14, n (%)	20,528 (49.3%)	268 (50.4%)	864 (51.1%)	5702 (50.6%)	13,694 (48.6%)	
≥15, n (%)	16,698 (40.1%)	209 (39.3%)	623 (36.8%)	4267 (37.9%)	11,599 (41.2%)	
missing, n (%)	690 (1.7%)	7 (0.5%)	26 (1.5%)	195 (1.7%)	462 (1.6%)	
Cohabitation in early pregnancy, n (%)	37,292 (89.5%)	462 (86.8%)	1507 (89.1%)	10,019 (88.9%)	25,304 (89.8%)	<0.05
missing, n (%)	2698 (6.5%)	38 (7.1%)	102 (6.0%)	772 (6.8%)	1786 (6.3%)	
Diabetic disease during pregnancy, n (%)	785 (1.9%)	26 (4.9%)	87 (5.1%)	373 (3.3%)	299 (1.1%)	<0.05
Smoking pregnancy week 32, n (%)	2752 (6.6%)	35 (6.6%)	151 (8.9%)	848 (7.5%)	1718 (6.1%)	<0.05
missing, n (%)	1395 (3.3%)	22 (4.1%)	75 (4.4%)	372 (3.3%)	926 (3.3%)	
**Infant Characteristics**						
Birthweight in standard deviation score^b^, mean (SD)	0.28 (0.99)	0.22 (0.98)	0.28 (0.97)	0.31 (1.01)	0.27 (0.98)	<0.05
Birthweight <10^th^ percentile^b^, n (%)	2109 (5.1%)	37 (7.0%)	83 (4.9%)	589 (5.2%)	1400 (5.0%)	
Birthweight <3^rd^ percentile^b^, n (%)	635 (1.5%)	13 (2.4%)	36 (2.1%)	185 (1.6%)	401 (1.4%)	
Birth length in standard deviation score^b^, mean (SD)	0.36 (1.01)	0.00 (0.98)	0.12 (0.93)	0.29 (0.99)	0.41 (1.01)	<0.05
Male gender, n (%)	20,785 (49.9%)	267 (50.2%)	858 (50.7%)	5621 (49.9%)	14,039 (49.8%)	0.92
Apgar score at 5 min						<0.05
0–6, n (%)	333 (0.8%)	34 (6.4%)	42 (2.5%)	77 (0.7%)	180 (0.6%)	
7–10, n (%)	41,055 (98.5%)	493 (92.7%)	1633 (96.5%)	11,110 (98.6%)	27,819 (98.7%)	
missing, n (%)	281 (0.7%)	5 (0.94%)	17 (1.0%)	84 (0.7%)	175 (0.6%)	
No breastfeeding at age two months, n (%)	1298 (3.1%)	26 (4.9%)	77 (4.6%)	392 (3.5%)	803 (2.9%)	<0.05
missing, n (%)	15,558 (37.3%)	208 (39.1%)	644 (38.1%)	4291 (38.1%)	10,415 (37.0%)	

^a^Asia, Africa, South America, former Soviet Union, Oceania.

^b^Birthweight and birth length for gestational age and sex according to the Swedish reference for birthweight, mean in standard deviation scores^35^.

The percentage of missing values across the independent variables varied between 0 and 37%. In total, 18,694 (45%) children had incomplete records. Breastfeeding at two months had 15,558 (37%) incomplete records, followed by maternal BMI with 4403 (10%) incomplete records. Maternal BMI and breastfeeding at two months were simultaneously missing in 1482 (4%) children and maternal smoking and breastfeeding were missing in 630 (2%) children.

**Figure 1:**
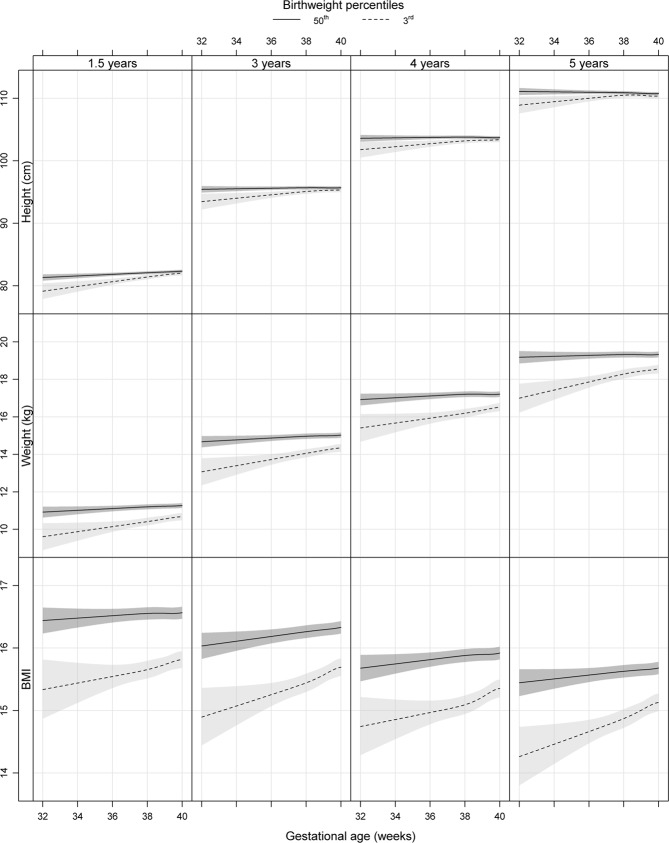
Estimated mean height, weight and BMI in children born standardized approptiate for gestational age (sAGA, birthweight 50^th^ percentile) and standardized small for gestational age (sSGA, birthweight 3^rd^ percentile) at age 1.5, 3, 4 and 5 years. Model adjusted for maternal height, BMI, diabetic disease, smoking habits at gestational week 32, country of birth, breastfeeding at age two months and gender. Significant differences are indicated by overlapping confidence intervals, which are presented in Tables 2 and 4.

Table 2 and Fig. 1 illustrate differences in estimated mean height and weight at ages 1.5 and 5 years between children born *s*AGA (birthweight 50^th^ percentile) or *s*SGA (birthweight 3^rd^ percentile) at gestational week 32 + 0 and 35 + 0 and children born *s*AGA at week 40 + 0 (reference). At 1.5 years, children born *s*AGA at week 32 + 0 or 35 + 0 were shorter compared with children born *s*AGA at 40 + 0 weeks, but estimated heights were comparable between the groups from 3 years of age. At 1.5 years, children born *s*SGA at week 32 + 0 and 35 + 0 were both shorter and lighter than the reference group, with a pattern of greater differences the more preterm born. Children born *s*SGA at 32 + 0 were 3 cm shorter and 1.5 kg lighter at 1.5 years, corresponding to −1.1 SD and −1.4 SD in height and weight, compared with children born *s*AGA at 40 + 0 (reference). The differences in estimated height and weight between *s*SGA at 32 + 0 or 35 + 0 and the reference group remained significant over the follow-up period of 5 years.

**Table 2:** Differences in estimated mean height and weight.

	Height difference^a^		Weight difference^a^	
	In cm (95% CI)	In SD (95% CI)	In kg (95% CI)	In SD (95% CI)
**1**.**5 years**				
**Born** ***s*** **AGA**				
32 + 0	−1.02 (−1.47; −0.57)	−0.36 −(0.53; −0.20)	−0.35 (−0.61; −0.09)	−0.29 (−0.51; −0.08)
35 + 0	−0.64 (−0.87; −0.40)	−0.23 (−0.31; −0.14)	−0.21 (−0.34; −0.08)	−0.18 (−0.28; −0.07)
**Born** ***s*** **SGA**				
32 + 0	−3.20 (−4.43; −1.97)	−1.14 (−1.58; −0.70)	−1.66 (−2.37; −0.95)	−1.38 (−1.98; −0.79)
35 + 0	−2.06 (−2.68; −1.45)	−0.74 (−0.96; −0.52)	−1.26 (−1.61; −0.91)	−1.05 (−1.34; −0.76)
40 + 0	−0.33 (−0.60; −0.06)	−0.12 (−0.21; −0.02)	−1.05 (−1.42; −0.68)	−0.50 (−0.63; −0.37)
**5 years**				
**Born** ***s*** **AGA**				
32 + 0	0.33 (−0.17; 0.84)	0.08 (−0.04; 0.19)	−0.15 (−0.45; 0.14)	−0.07 (−0.2; 0.06)
35 + 0	0.25 (−0.01; 0.51)	0.06 (−0.00; 0.12)	−0.08 (−0.23; 0.07)	−0.03 (−0.1; 0.03)
**Born** ***s*** **SGA**				
32 + 0	−1.84 (−3.16; −0.53)	−0.42 (−0.72; −0.12)	−2.33 (−3.09; −1.58)	−1.01 (−1.34; −0.69)
35 + 0	−1.02 (−1.68; −0.37)	−0.23 (−0.38; −0.08)	−1.69 (−2.06; −1.31)	−0.73 (−0.9; −0.57)
40 + 0	−0.37 (−0.67; −0.07)	−0.08 (−0.15;− 0.02)	−1.61 (−2.01; −1.20)	−0.34 (−0.41; −0.26)

Children born standardized appropriate for gestational age (*s*AGA) or small for gestational age (*s*SGA) in gestational weeks 32 + 0, 35 + 0 and 40 + 0 compared with children born *s*AGA in gestational week 40 + 0.

*s*AGA defined as birthweight 50^th^ percentile and *s*SGA as birthweight 3^rd^ percentile of expected for gestational age and sex according to the Swedish reference standards for birthweight, respectively^35^.

^a^Model adjusted for maternal height, BMI, country of birth, maternal diabetic disease during pregnancy, smoking in pregnancy week 32 and breastfeeding at age two months.

**Figure 2:**
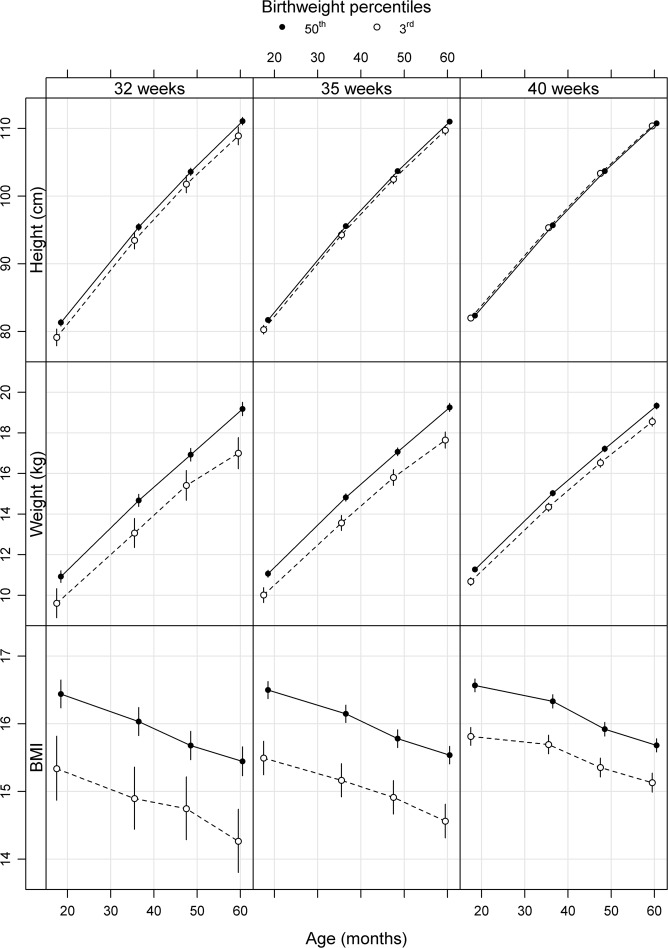
Estimated mean height, weight and BMI from age 1.5 to 5 years in children born standardized approptiate for gestational age (sAGA, birthweight 50^th^ percentile) and standardized small for gestational age (sSGA, birthweight 3^rd^ percentile) in gestational week 32 + 0, 35 + 0 and 40 + 0. Model adjusted for maternal height, BMI, diabetic disease, smoking habits at gestational week 32, country of birth, breastfeeding at age two months and gender. Significant differences are indicated by overlapping confidence intervals, which are presented in Tables 3 and 4.

Table 3 and Fig. 2 illustrate differences in the estimated height and weight at the same follow-up ages, but children born *s*AGA (birthweight 50^th^ percentile) and *s*SGA (birthweight 3^rd^ percentile) at the same gestational age are compared. In comparison with children born *s*AGA at week 32 + 0, 35 + 0 and 40 + 0, those born *s*SGA at the corresponding gestational age had shorter estimated mean heights at all follow-up ages. There was a trend of more pronounced differences in children born in week 32 + 0 than 35 + 0, which lasted over the follow-up period. A pattern of larger differences the shorter the gestational age was seen.

**Table 3:** Differences in estimated mean height and weight.

	Height difference^a^		Weight difference^a^	
	In cm (95% CI)	In SD (95% CI)	In kg (95% CI)	In SD (95% CI)
**1**.**5 years**				
**Born** ***s*** **SGA**				
32 weeks	−2.18 (−3.49; −0.87)	−0.78 (−1.25; −0.31)	−1.31 (−2.06; −0.56)	−1.09 (−1.72; −0.47)
35 weeks	−1.43 (−2.07; −0.78)	−0.51 (−0.74; −0.28)	−1.05 (−1.42; −0.68)	−0.88 (−1.18; −0.57)
40 weeks	−0.33 (−0.60; −0.06)	−0.12 (−0.21; −0.02)	−0.60 (−0.75; −0.44)	−0.50 (−0.63; −0.37)
**5 years**				
**Born** ***s*** **SGA**				
32 weeks	−2.18 (−3.60; −0.75)	−0.50 (−0.82; −0.17)	−2.18 (−3.00; −1.36)	−0.95 (−1.30; −0.59)
35 weeks	−1.27 (−1.97; −0.57)	−0.29 (−0.45; −0.13)	−1.61 (−2.01; −1.20)	−0.70 (−0.87; −0.52)
40 weeks	−0.37 (−0.67; −0.07)	−0.08 (−0.15; −0.02)	−0.78 (−0.95; −0.60)	−0.34 (−0.41; −0.26)

Children born standardized small for gestational age (*s*SGA) in gestational weeks 32 + 0, 35 + 0 and 40 + 0 compared with children born standardized appropriate for gestational age (*s*AGA) in the corresponding gestational week.

*s*AGA defined as birthweight 50^th^ percentile and *s*SGA as birthweight 3^rd^ percentile of expected for gestational age and sex according to the Swedish reference for birthweight^35^.

^a^Model adjusted for maternal height, BMI, country of birth, maternal diabetic disease during pregnancy, smoking in pregnancy week 32 and breastfeeding at age two months.

Table 4, Figs 1 and 2 illustrate the ratio of estimated mean BMI at ages 1.5 and 5 years. Children born *s*AGA (birthweight 50^th^ percentile) of all gestational ages had comparable BMIs throughout the follow-up period. Children born *s*SGA (birthweight 3^rd^ percentile) of all gestational ages had lower estimated mean BMI at all follow-up ages compared with children born *s*AGA at 40 + 0. At 5 years, children born *s*SGA at 32 + 0, 35 + 0 and 40 + 0 had 9%, 7% and 4% lower estimated mean BMI than those born *s*AGA at 40 + 0, respectively. Also if we compared children born *s*AGA and *s*SGA of the same gestational age, children born *s*SGA had lower estimated mean BMI at age 5 years than those born *s*AGA.

**Table 4:** Ratio of estimated mean BMI in children born standardized appropriate for gestational age (*s*AGA) or small for gestational age (*s*SGA) in week 32 + 0 (*s*AGA 32 and *s*SGA 32), week 35 + 0 (*s*AGA 35 and *s*SGA 35) or week 40 + 0 (*s*AGA 40 and *s*SGA 40).

	Adjusted BMI ratio^a^	
	1.5 years	5 years
*s*AGA 32/*s*AGA 40	0.99 (0.98; 1.00)	0.98 (0.97; 1.00)
*s*AGA 35/*s*AGA 40	1.00 (0.99; 1.00)	0.99 (0.98; 1.00)
*s*SGA 32/*s*AGA 40	0.93 (0.90; 0.95)	0.91 (0.88; 0.94)
*s*SGA 35/*s*AGA 40	0.94 (0.92; 0.95)	0.93 (0.91; 0.94)
*s*SGA 40/*s*AGA 40	0.95 (0.95; 0.96)	0.96 (0.96; 0.97)
*s*SGA 32/*s*AGA 32	0.93 (0.90; 0.96)	0.92 (0.89; 0.96)
*s*SGA 35/*s*AGA 35	0.94 (0.92; 0.95)	0.94 (0.92; 0.95)

*s*AGA defined as birthweight 50^th^ percentile and *s*SGA as birthweight 3^rd^ percentile of expected for gestational age and sex according to the Swedish reference for birthweight^35^.

^a^Model adjusted for maternal height, BMI, country of birth, maternal diabetic disease during pregnancy, smoking in pregnancy week 32 and breastfeeding at age two months.

**Figure 3:**
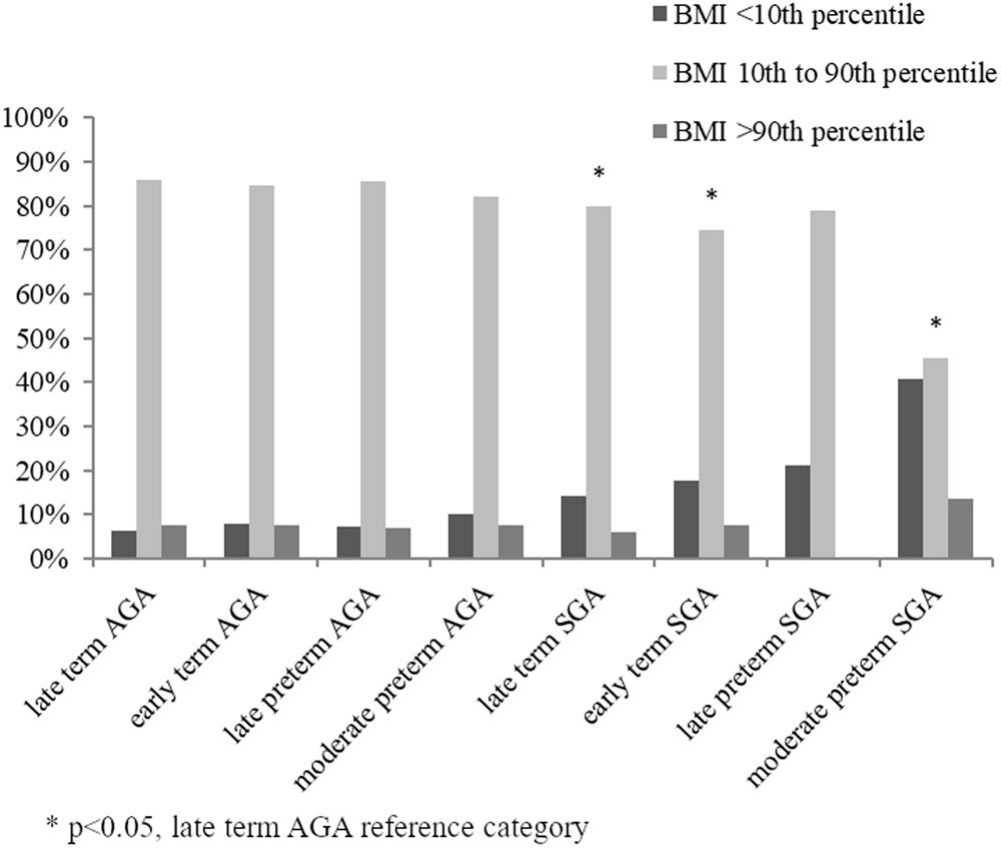
Proportion of children with low, normal and high BMI.

At 5 years of age, the proportion of children with a normal BMI (10^th^–90^th^ percentile) varied according to SGA-status and gestational age at birth, see Fig. 3. Children born SGA (<10^th^ percentile) and moderate preterm (32–34 weeks) less often had a normal BMI compared with children born AGA (10^th^–90^th^ percentile) and late term (39–40 weeks) (p < 0.05). There was a J-shaped pattern among children born SGA and moderate preterm with a tendency of more subjects with high BMI, but even more children with low BMI at 5 years.

## Conclusions

### Statement of principal findings

In children born moderate to late preterm we could show a difference in postnatal growth depending on coexistence of SGA birth or not. Children born AGA and moderate to late preterm had comparable estimated mean height, weight and BMI as children born AGA and term after 3 years of age. Children born SGA and moderate to late preterm were shorter and lighter and had a lower BMI than children born AGA and term throughout the whole follow-up period of 5 years. There was a pattern of larger differences with decreasing gestational age at birth. At age 5 years, fewer children born SGA and moderate preterm had a normal BMI compared with those born AGA and late term.

### Strengths and limitations

The main strength of the study was the large cohort size of 41,669 children. The cohort can be considered as population based, including all deliveries and child health centres in one county of Sweden. Antenatal, delivery and child health care is standardized and free of charge in Sweden, and inhabitants in Uppsala County have very high attendance to child health care^18^. The size of the cohort allowed us to divide the cohort into narrow strata of gestational age, and also use the 3^rd^ percentile as definition of SGA in the GLS model. Children born very preterm is a group with high co-morbidity. By excluding them, we could reduce the risk of exaggerating the potential impact of moderate to late preterm birth on postnatal growth and body proportions, often called type I error. Multiple pregnancy has a high level of co-morbidity and is one of the most common etiological factors behind moderate to late preterm birth^19^. Therefore, we also excluded children born in multiple pregnancies. Anthropometric data was prospectively collected by a trained nurse, which minimizes the risk of recall and information bias. The linkage of data from several population based registries with information on socio-economic confounders as well as maternal and infant characteristics made it possible to adjust for several important confounders.

There are some important limitations of the study. First, despite the large cohort size, there were relatively few children born moderate preterm and SGA. Considering this, we could not calculate the proportion of children with a normal BMI at 5 years and use of the 3^rd^ percentile as cut-off. Second, data on some important potential confounders, most importantly breastfeeding, was incomplete. In order to use the whole dataset for the analyses, we imputed data on breastfeeding. Third, in order to guarantee the integrity of the study subjects, we did not have data on date of birth. Hence, we were not able to correct the age, at which anthropometric measurements were performed, for degree of prematurity. This might have an implication on the results, especially at age 1.5 years. However, any underestimation of childhood body size in children born preterm should be similar for those born SGA and AGA. Fourth, we lack data on paternal height, which influences growth in early childhood equally as maternal height^20^. Last, we did not have data on paternal BMI or eating habits of the families, which might be an important confounder for body proportion during childhood.

### Comparison with earlier studies

In a Dutch study, they followed a cohort of comparable size to ours regarding growth in height and weight until four years of age^21,22^. In the first paper of the study, they showed that without considering birthweight, children born in week 32–35 were shorter and lighter at 4 years than those born term. Moreover, among children born moderate preterm, those born SGA had an increased risk of short stature at 4 years compared with those born AGA. The second paper examined combined exposure to being born SGA or preterm (week 26–35). In conformity with our results, combined exposure had larger impact on growth in height and weight than isolated exposure. In the Dutch study, each study subject was measured on average 9.9 times. It is possible that largely deviant growth in a few subjects have large impact on the cohort mean as they did not account for repeated measurements. In our study we constructed a GLS model, where gestational age and standardized birthweight as well as postnatal anthropometrics were used as continuous variables. This allowed us to construct a prediction model with smoothed curves and more precise estimated means, which accounts for repeated measurements. Despite the different statistical methods, the results of the Dutch and our study point in the same direction and thus show reproducibility.

We found of a J-shaped pattern in BMI at 5 years in children born SGA and moderate preterm, with a tendency towards increased incidence of obesity. A large proportion had low BMI, which generates a low mean weight. This is in accordance with earlier studies of body proportions in adulthood in children born SGA or preterm, especially for children with short adult stature^23,24^. At 5 years of age, catch-up growth after being born SGA has usually already taken place^3^. Considering this, our finding of a shorter mean height of 2 cm at 5 years in children born SGA and moderate preterm might have important cognitive implications as more children will remain short. Even though evidence is limited for children born moderate preterm, the risk of adverse health outcome after rapid catch-up in weight or limited catch-up in height in early childhood indicates that postnatal growth patterns are of importance, and more research is needed in children born moderate preterm^25,26^.

In our study, we chose to use birthweight reference standards when we estimated birthweight for gestational age and sex. These references tend to underestimate the frequency of SGA in children born preterm^27^. Thereby, the difference between children born AGA and SGA might be underestimated.

### Perspectives

After combined exposure to being born SGA and moderate to late preterm, body proportions differed from children with single exposure to SGA or preterm birth. As BMI is a measurement that includes height as well as weight, this shows that combined exposure not merely increases the risk of a slower growth, but also has an impact on body proportions. With height being largely genetically and growth hormone dependent, the growth hormone axis does not seem to be heavily influenced. However, we speculate that a J-shaped pattern of BMI at 5 years of age after being born SGA and moderate preterm might have an impact on metabolic and cardiovascular future health.

Andrews *et al*. showed that in children born very preterm, postnatal growth restriction can be less pronounced with improved early nutritional care^28^. Even though children born after week 32 suffer from less neonatal morbidity than those born very preterm, early feeding difficulties are still common. Fewer infants born preterm are breast-fed compared with those born term^29,30^. Although we do not known if feeding-practices in children born moderate preterm affect postnatal growth, we speculate that improved nutrition might limit neonatal growth restriction and, if born SGA, early catch-up growth might be enhanced.

The potential metabolic risks but cognitive advantages of catch-up growth render difficulties in recommendations of appropriate nutrition during early infancy. There is a consensus on overall benefits of catch-up growth in preterm infants, which is mainly based on studies on infants born before 31 weeks^4^. However, despite slower weight-gain, very preterm born infants have neurocognitive benefits from breastfeeding^31^. In our study, being born moderate to late preterm and SGA, but not AGA, was associated with changes in body proportions. Unfortunately, we had too few subjects with complete data to explore if breastfeeding seemed to modify growth patterns. More research is needed to evaluate if postnatal growth patterns and long-term metabolic outcomes are affected by breastfeeding habits in children born moderate preterm^32^.

In conclusion, compared with being born AGA and term, being born SGA and moderate to late preterm is associated with shorter stature and lower BMI during the first five years of life. SGA status was found to have a greater impact on growth and body proportions than gestational age at birth. Thus, in the perspective of postnatal growth, children with a satisfactory intrauterine growth seem to cope with moderate to late preterm birth well. For children with insufficient intrauterine growth, there is a pattern of interaction with shorter stature and more abnormal body proportions the shorter gestational age.

## Methods

### Data sources

Uppsala County Mother and Child Database is a population based database which contains data from several national and regional registries. The included registries are the Medical Birth Register (MBR), Database of statistics of the Child Health Care Unit in Uppsala County, Register of Education and Register of Total Population. All children born at Uppsala University Hospital, the only hospital with a delivery unit in the county, who have later visited child health care in Uppsala County, Sweden, during the years 2000–2015 are included in the Uppsala County Mother and Child Database.

The MBR is a national registry and contains information on maternal characteristics, pregnancy complications, delivery, neonatal characteristics and complications of all pregnancies and births after 22 gestational weeks. It is based on mandatory registration of prospectively collected information. In Sweden, maternal, delivery and child health care are free of charge and follows a standardized schedule. The first antenatal visit usually takes place before 13 weeks of pregnancy, and involves an interview about medical and obstetric history, social situation including cohabitation and smoking habits three months before pregnancy and in early pregnancy^33^. The woman is weighed and her height is recorded. In pregnancy week 32, the woman is once again asked about her current smoking habits. Data is recorded using check-boxes, and anthropometric measurements are recorded in absolute numbers. Complications and diseases are classified using the International Classification of Diseases (ICD).

Data from child health care in Uppsala County is registered in the Database of statistics of the Child Health Care Unit in Uppsala County. 97% of children living in Uppsala County regularly visit child health services during their first six years^18^. The registry contains information on anthropometric measurements and health data, as well as data from parental interviews about exclusive or partial breastfeeding at two months, and smoking habits at 4 months of age.

Data on maternal level of education is collected from the Register of Education, and maternal country of birth from the Register of Total Population. We used personal identification number assigned to all citizens in Sweden to cross-link data between the registries^34^.

### Subjects

Uppsala County Mother and Child Database contains information on 57,044 children born in Uppsala County. All children had known sex. We excluded children with unknown gestational age (n = 64) or birthweight (n = 54). A birthweight of <−5 or >5 standard deviations (SD) of expected birth weight for gestational age and sex was considered misclassified and hence excluded (n = 27). Further we excluded children born after multiple pregnancies (n = 1629), with chromosome aberrations or malformations (n = 956), leaving 54,314 children in the study population. Since the aim was to study children with moderate to late preterm birth, we excluded children born with gestational age <32 weeks (n = 312) and ≥41 weeks (n = 12,333). The final cohort consisted of 41,669 children.

### Exposures

The studied exposures were gestational age and weight for gestational age and sex at birth. Gestational age was expressed as completed gestational weeks and days at birth. In the first model, gestational age was used as a continuous variable, and the outcome was estimated for children born with gestational age 32 + 0, 35 + 0 and 40 + 0. Next, the cohort was stratified according to gestational age as moderate preterm (32–34 weeks), late preterm (35–36 weeks), early term (37–38 weeks) and late term (39–40 weeks). Most pregnancies, >92%, were dated with early second trimester ultrasound.

Birthweight for gestational age and sex was calculated using the national reference curve for birthweight^35^. We used two different definitions of being born SGA. First, with birthweight as a continuous exposure variable, where birthweight for gestational age and sex at the 3^rd^ percentile was defined as standardized SGA (*s*SGA). Second, we classified SGA as birthweight <10^th^ percentile of expected.

### Outcome

The main outcome was growth in height (cm), weight (kg) and BMI (kg/m^2^) during the first five years of childhood. Height and weight were measured at ages 1.5, 3, 4 and 5 years by a trained nurse in routine child health care. Since 2005, anthropometrics are registered electronically, thus data is missing for the younger ages in children born before 2003. Only measurements performed within 2 months from planned according to the age of the child are included in the Uppsala County Mother and Child Database.

### Covariates

Maternal age, height, BMI, parity, parental cohabitation at first antenatal visit, maternal diabetic disease during pregnancy, smoking habits in pregnancy week 32, maternal country of birth, maternal level of education, male gender, Apgar score at 5 min and exclusive or partial breastfeeding at age two months were considered potential covariates. Maternal diabetic disease was identified by the corresponding ICD-10 codes (O241-O243, E10-14).

In order to select covariates for the multivariable models, we drew a directed acyclic graph (DAG). The minimal sufficient set for confounder adjustment included maternal height, BMI, diabetic disease, smoking habits at gestational week 32, country of birth and breastfeeding at age two months.

### Statistical analysis

Clinical and demographic characteristics of the study population were cross-tabulated according to gestational age at birth, grouped as moderate preterm (32–34 weeks), late preterm (35–36 weeks), early term (37–38 weeks) and late term (39–40 weeks) birth. Children born late term were treated as reference group.

In order to account for the repeated measures in the outcome, we applied a generalized least squares model (GLS). Unlike generalized mixed effects model, GLS does not use random effects, but instead requires a specification of the within individual correlation structure. The regression model included the anthropometric measurements at each visit to child health care (age 1.5, 3, 4 and 5 years), but also the interaction between gestational age and standardized birthweight at birth. The model was adjusted for gender and potential confounders suggested by the DAG. All continuous variables were modelled using restricted cubic splines with 3–5 knots based on the apparent strength of association with the outcome. All models assumed a continuous autoregressive AR(1) correlation structure between the repeated measurements.

The GLS model described the growth trajectories according to standardized birthweight and gestational age. Using the GLS model, we estimated means of height, weight and BMI, both for children with birthweight 50^th^ percentile of expected for gestational age and sex (standardized AGA, *s*AGA) and 3^rd^ percentile (standardized SGA, *s*SGA) at gestational week 32 + 0, 35 + 0 and 40 + 0. We calculated the contrasts between estimated mean heights and mean weights with 95% confidence interval (CI), i.e. the difference between children born *s*AGA or *s*SGA at 32 + 0, 35 + 0 or 40 + 0 gestational weeks and the reference value (*s*AGA week 40 + 0). Next, we compared children born *s*AGA with those born *s*SGA in the same gestational week. The contrasts were calculated as difference in cm or kg, but also in standard deviations according to the Swedish reference curve for height and weight^36^.

The contrast of estimated mean BMI was calculated as the ratio between the estimated mean BMI and the reference value of estimated mean BMI, e.g. a ratio of 0.95 indicates that the BMI was 5% lower than the reference value. First, we compared children born *s*AGA or *s*SGA in gestational week 32 + 0, 35 + 0 or 40 + 0, with the reference *s*AGA week 40 + 0. In the next step, sSGA at 32 + 0, 35 + 0 and 40 + 0 weeks were compared with *s*AGA in the corresponding gestational week (reference value).

Incomplete baseline variables were imputed under fully conditional specification^37^. The outcome values were included in the imputation, but the imputed outcome values were subsequently discarded^38^. Model parameters were estimated in each imputed data set separately and their estimates and standard errors were combined using Rubin’s rules. For comparison, a complete case analysis was also performed.

Lastly, we classified the cohort according to birthweight, as AGA or SGA, and gestational age (late term, early term, late preterm and moderate preterm). We used the anthropometric measurements at age 5 years to calculate the proportion of children with normal BMI and compared the groups using Chi-Square test.

IBM SPSS Statistics 2.5 and R version 3.5.0 using the mice and rms packages were used in the statistical calculations.

### Research involving human participants

Ethic permission was approved by the Regional Ethical Review Board in Uppsala (no. 2012/410). All procedures involving human subjects were carried out in accordance with the ethical standards of the 1964 Helsinki declaration. All registry data was merged and de-identified by Statistics Sweden. Thereby, informed consent was not required.

## Data Availability

The datasets generated during and/or analyzed during the current study are not publicly available due to the ethical and legal restrictions prohibiting the sharing of personal data, but are available from the corresponding author on reasonable request.
